# Supplementary material for: Continuous remote monitoring of postoperative vital signs with the Biobeat patch: A multicenter prospective observational study
Source: PLoS One. 2026 Apr 20;21(4):e0344662. doi: 10.1371/journal.pone.0344662 (PMC13094953; doi:10.1371/journal.pone.0344662)
Supplement: S1 Appendix — (PDF) [file pone.0344662.s001.pdf]

Protocol-Summary-French (original)
and English translation

SYNOPSIS

IDENTIFICATION DE L’ESSAI CLINIQUE

Numéro de code promoteur : 2020_0050
Version et date : Version n°4 du 03/12/2021

Titre de l’essai : Dépistage des complications postopératoires, en particulier hémodynamiques par le monitorage continu utilisant le patch Biobeat

Investigateur Coordonnateur : **Dr Alexis PATERNOT**

Multicentrique :<br>Centre 1 : Hôpital Foch, Suresnes<br>Centre 2 : Hôpital Saint-Joseph, Paris<br>Centre 3 : Hôpital Diaconesses Croix Saint-Simon, Paris	Nombre de patients : 114

IDENTIFICATION DU PROMOTEUR

HOPITAL FOCH – DELEGATION A LA RECHERCHE CLINIQUE (DRCI)
Contact : Mme Elisabeth HULIER-AMMAR
Tél : 00 33 1 46 25 11 75 / e-mail : e.hulier-ammar@hopital-foch.com

RATIONNEL DE L’ETUDE

La mortalité postopératoire reste une réalité comme l’a montré l’étude de l'International Surgical Outcomes Study publiée en 2016 (International Surgical Outcomes Study 2016) ou celle de Turan et coll. (Turan, Chang et al. 2019), plus centrée sur l’évolution de la pression artérielle. En effet, les complications postopératoires sont courantes dans les services de chirurgie et passent souvent inaperçues.

L’évolution technologique est marquée par la possibilité de surveiller les paramètres vitaux à l’aide de patchs simplement collés sur la peau et connectés sans fil vers un site de stockage des données avant leur analyse (Khanna, Hoppe et al. 2019). L'emploi de ces nouveaux dispositifs offrirait une solution d'alerte plus précoce et plus complète d'une dégradation de l'état clinique.

Biobeat Technologies Ltd (Petah Tikva, Israël ; http://www.bio-beat.com) a développé un capteur portable, patch cutané ou montre, qui comprend une application mobile. Le capteur enregistre en continu l’onde photopléthysmographique qui permet de calculer plusieurs paramètres physiologiques : la fréquence cardiaque (FC), la SPO2, la pression artérielle systolique (PAS) et la variation de la PAS, la pression artérielle diastolique (PAD) et la variation de la PAD, le volume systolique (VS), le débit cardiaque (CO), l'index cardiaque (IC), la résistance vasculaire systémique (RVS), et la fréquence respiratoire (RR).

L’objet de cette étude est le recueil est la quantification des anomalies hémodynamiques, respiratoire et de température détectée par un monitoring usuel (suivi infirmier) et un monitoring en continu par le patch Biobeat.

INFORMATIONS GENERALES SUR L’ESSAI

Indications : Patients devant subir une intervention chirurgie lourde (chirurgie digestive, gynécologique ou urologique) pour laquelle une surveillance des constantes vitales post-opératoire est indiquée.

Méthodologie : Recueil des données de monitoring issues du suivi infirmier habituel et du suivi continu avec le patch Biobeat, comparaison de la fréquence des évènements cliniques observés.

Objectif principal :
L'objectif principal est d'estimer la proportion de patients présentant une hypotension sévère et notamment de corroborer la prévalence trouvée dans Liem et al. [Postoperative Hypotension after Noncardiac Surgery and the Association with Myocardial Injury. Anesthesiology 2020], soit 8%.

Objectifs secondaires :

1. Quantifier et comparer la fréquence des anomalies hémodynamique postopératoires détectée par le suivi infirmier et le patch Biobeat
2. Quantifier et comparer la fréquence des anomalies respiratoires postopératoires détectée par le suivi infirmier et le patch Biobeat
3. Quantifier et comparer la fréquence des anomalies de la température détectée par le suivi infirmier et le patch Biobeat
4. Déterminer la fréquence des artefacts et des périodes d'absence de données du dispositif médical
5. Recueil des complications postopératoires survenues durant la période de surveillance
6. Evaluer la tolérance des patients quant au port du dispositif Biobeat

Critères d'inclusion :

a. Patient âgé de 18 ans ou plus
b. Patient devant subir une intervention chirurgicale majeure digestive, gynécologique, orthopédique ou urologique (durée prévisible d'intervention supérieure à 2 heures) pour laquelle une surveillance des constantes vitales post-opératoire est indiquée
c. Durée d'hospitalisation postopératoire prévisible ≥ 2 nuits
d. Patient affilié à un régime d'Assurance Maladie.
e. Ne pas s'être opposé à participer à la recherche

Critères de non inclusion :

a. Patient présentant une déformation importante, un gonflement, une irritation ou présentant une infection localisée, une ulcération ou des lésions cutanées au niveau du torse.
b. Patient portant un implant électronique sous-cutané de type pacemaker.
c. Patient ayant un scanner ou une IRM déjà prévu pendant les trois premiers jours post-opératoires
d. Patient souffrant de tremblements ou de convulsions
e. Patient présentant un tatouage au niveau du torse
f. Patient présentant une pilosité importante au niveau du torse
g. Patient présentant une allergie connue aux métaux, au plastique et au silicone
h. Patient privé de liberté ou sous tutelle
i. Grossesse ou allaitement

Critère d'exclusion :

j. Réaction cutanée importante en réaction au dispositif médical

ORGANISATION DE L'ETUDE

Participation des personnes :

Sélection et inclusion

Après vérification des critères d’inclusion et de non inclusion, le médecin informe le patient de l’étude et s’assure de sa non opposition à participer. Les données démographiques, l’histoire de la maladie et les traitements concomitants seront recueillis

Suivi post-opératoire

- Recueil des données issues du suivi infirmier à une fréquence définie par l’équipe médicale : pression artérielle, fréquence cardiaque, fréquence respiratoire, saturation en oxygène, température
- Pose du patch Biobeat et recueil des données cliniques (pression artérielle, fréquence cardiaque, fréquence respiratoire, saturation en oxygène, température) en continu

Visite de fin d’étude :

- Arrêt du recueil des données cliniques du suivi infirmier
- Retrait du patch Biobeat
- Remplissage du questionnaire de tolérance du dispositif Biobeat par les soignants et le patient

CONSIDERATIONS STATISTIQUES

Calcul du nombre de patients nécessaires :

L’objectif principal est d'estimer la proportion de patients présentant une hypotension sévère et notamment de corroborer la prévalence trouvée dans Liem et al. [Postoperative Hypotension after Noncardiac Surgery and the Association with Myocardial Injury. Anesthesiology 2020], soit 8%. Pour obtenir une précision de 10 % (± 5 %) avec un risque alpha bilatéral de 5 %, 114 patients devraient être inclus.

Populations d’analyse :

Deux populations seront prises en compte dans l’analyse :

- ✓ En intention de traiter : qui comprendra tous les patients a inclus pour lesquels un CRF a été complété.
- ✓ Per protocole : tous les patients non perdus de vue pour lesquelles la visite de fin d’étude aura été effectuée.

Méthode d’analyse statistique :

La population d’analyse sera décrite avec des fréquences et pourcentages pour les variables à caractère qualitatif et des moyennes et écarts-types ou médianes et écarts interquartiles pour les variables à caractère quantitatif selon leur distribution.

Tous les tests effectués seront bilatéraux et le risque d’erreur de première espèce α fixé à 5%. Les analyses seront réalisées sur le logiciel SAS® 9.4

DUREE PREVUE DE L’ESSAI

Période d’inclusion : 24 mois

Période de participation à l’étude pour un patient : 3 jours maximum

Durée globale de l’essai période de suivi incluse : 24 mois et 3 jours maximum

ASSURANCE QUALITE

Recueil des données :

Les données recueillies à partir du dossier médical pour chaque patient seront les suivantes :

- ✓ Les données démographiques
  - date de naissance (mois et année)
  - sexe
- ✓ Le type d'intervention
- ✓ Les antécédents médicaux significatifs
- ✓ Les traitements médicaux en cours
- ✓ Recueil des complications postopératoires
- ✓ Recueil des effets indésirables
- ✓ Satisfaction des patients quant au port du dispositif Biobeat

Gestion des données

Les patients seront identifiés dans l'étude par un numéro d'inclusion.

Les données seront saisies dans un eCRF sécurisé.

Les données collectées par les patchs Biobeat transiteront sur un serveur Hébergement agréé données santé (HADS).

Contrôle Qualité :

L'équipe de la Délégation à la Recherche Clinique et à l'Innovation (DRCI) de l'hôpital Foch sera en charge du suivi du contrôle qualité de l'étude.

Un Attaché de Recherche Clinique (ARC) de l'hôpital Foch, Promoteur, sera chargé du suivi de l'étude, de la vérification des non-oppositions, du contrôle qualité des données recueillies…

REGLEMENTAIRE

Comité de Protection des Personnes – CPP Ile-de-France II : Avis favorable obtenu le 29/09/2020

CNIL : Engagement à la Méthodologie de Référence 003 (MR003) le 06/02/2017

SYNOPSIS

CLINICAL TRIAL IDENTIFICATION
Sponsor code number: 2020_0050<br>Version and date: Version n°4 dated 03/12/2021<br><br>Trial title: Screening for postoperative complications, particularly hemodynamic complications, by continuous monitoring using the Biobeat patch<br><br>Coordinating Investigator: Dr Alexis PATERNOT<br><br>Multicenter: Number of patients: 114<br>Center 1: Hôpital Foch, Suresnes<br>Center 2: Hôpital Saint-Joseph, Paris<br>Center 3: Hôpital Diaconesses Croix Saint-Simon, Paris

PROMOTER IDENTIFICATION
**HOPITAL FOCH - DELEGATION A LA RECHERCHE CLINIQUE (DRCI)**<br>Contact: Mme Elisabeth HULIER-AMMAR<br>Tel: 00 33 1 46 25 11 75 / e-mail: e.hulier-ammar@hopital-foch.com

RATIONALE OF THE STUDY
Postoperative mortality remains a reality, as shown by the International Surgical Outcomes Study published in 2016 (International Surgical Outcomes Study 2016) or that of Turan et al. (Turan, Chang et al. 2019), more focused on the evolution of blood pressure. Indeed, postoperative complications are common in surgical departments and often go unnoticed.<br><br>The technological evolution is marked by the possibility of monitoring vital parameters using patches simply glued to the skin and connected wirelessly to a data storage site prior to analysis (Khanna, Hoppe et al. 2019). The use of these new devices would offer an earlier and more comprehensive warning solution of a deteriorating clinical condition.<br><br>Biobeat Technologies Ltd (Petah Tikva, Israel; http://www.bio-beat.com) has developed a wearable sensor, skin patch or watch, which includes a mobile app. The sensor continuously records the photoplethysmographic waveform, which is used to calculate several physiological parameters: heart rate (HR), SPO2, systolic blood pressure (SBP) and SBP variation, diastolic blood pressure (DBP) and DBP variation, stroke volume (SV), cardiac output (CO), cardiac index (CI), systemic vascular resistance (SVR), and respiratory rate (RR).<br><br>The purpose of this study is to collect and quantify hemodynamic, respiratory and temperature abnormalities detected by usual monitoring (nursing follow-up) and continuous monitoring using the Biobeat patch.

GENERAL TEST INFORMATION
Indications : Patients undergoing major surgery (digestive, gynecological or urological) for whom post-operative monitoring of vital constants is indicated.<br><br>Methodology: Collection of monitoring data from the usual nursing follow-up and continuous follow-up with the Biobeat patch, comparison of the frequency of clinical events observed.<br><br>Primary objective:<br>The main objective is to estimate the proportion of patients with severe hypotension, and in particular to corroborate the prevalence found in Liem et al. [Postoperative Hypotension after Noncardiac Surgery and the Association with Myocardial Injury. Anesthesiology 2020], i.e. 8%.<br>Secondary objectives:<br>1. Quantify and compare the frequency of postoperative hemodynamic abnormalities detected by nurse monitoring and the Biobeat patch.<br>2. Quantify and compare the frequency of postoperative respiratory abnormalities detected by nursing follow-up and the Biobeat patch.<br>3. To quantify and compare the frequency of temperature abnormalities detected by nursing follow-up and the Biobeat patch.<br>4. Determine the frequency of artifacts and periods of missing data from the medical device.<br>5. Collect postoperative complications occurring during the monitoring period<br>6. Evaluate patients' tolerance of the Biobeat device.<br><br>Inclusion criteria :<br>a. Patient 18 years of age or older<br>b. Patient scheduled for major digestive, gynecological, orthopedic or urological surgery (expected duration of surgery greater than 2 hours) for which post-operative vital signs monitoring is indicated.<br>c. Foreseeable post-operative hospital stay ≥ 2 nights<br>d. Patient affiliated to a health insurance scheme.<br>e. Did not object to participating in the research<br>Non-inclusion criteria:<br>a. Patient with significant deformity, swelling, irritation or localized infection, ulceration or skin lesions on the torso.<br>b. Patient with a pacemaker-type subcutaneous electronic implant.<br>c. Patient having a CT or MRI scan already scheduled during the first three days post-operatively<br>d. Patient suffering from tremors or convulsions<br>e. Patient with a tattoo on the torso<br>f. Patient with extensive chest hair growth<br>g. Patient with known allergy to metals, plastics or silicone<br>h. Patient deprived of liberty or under guardianship<br>i. Pregnancy or breast-feeding<br>Exclusion criteria:<br>j. Significant skin reaction to medical device

ORGANIZATION OF THE STUDY
Patient participation:<br>**Selection and inclusion**<br>After checking the inclusion and non-inclusion criteria, the physician informs the patient of the study and ensures that he/she is not opposed to participating. Demographic data, disease history and concomitant treatments will be collected.<br>**Post-operative follow-up**<br>• Collection of nursing follow-up data at a frequency defined by the medical team: blood pressure, heart rate, respiratory rate, oxygen saturation, temperature, etc.<br>• Placement of Biobeat patch and continuous collection of clinical data (blood pressure, heart rate, respiratory rate, oxygen saturation, temperature).<br>**End-of-study visit:**<br>• Stop collecting clinical data for nursing follow-up<br>• Removal of Biobeat patch<br>• Completion of Biobeat tolerance questionnaire by caregivers and patient

STATISTICAL CONSIDERATIONS
Calculation of number of patients required:<br>The main objective is to estimate the proportion of patients with severe hypotension and not to corroborate the prevalence found in Liem et al. [Postoperative Hypotension after Noncardiac Sur the Association with Myocardial Injury. Anesthesiology 2020], i.e. 8%. To obtain a precision of 5%) with a two-sided alpha risk of 5%, 114 patients should be included.<br>Analysis populations:<br>Two populations will be considered in the analysis:<br>• Intent-to-treat: which will include all patients a included for whom a CRF has been c<br>• Per protocol: all patients not lost to follow-up for whom the end-of-study visit performed.<br>Statistical analysis method:<br>The analysis population will be described using frequencies and percentages for c qualitative variables, and means and standard deviations or medians and interquartile ranges for c quantitative variables, according to their distribution.<br>All tests will be two-tailed, with the risk of first-species error α set at 5%. The tests will be performed using SAS® 9.4 software.

INTENDED DURATION OF THE TRIAL
Inclusion period: 24 months<br>Study participation period for a patient: 3 days maximum<br>Overall duration of the trial including follow-up period: 24 months and 3 days maximum

QUALITY ASSURANCE
Data collection :<br>The data collected from the medical record for each patient will be as follows:

• Demographic data<br>- date of birth (month and year)<br>- gender<br>• Type of procedure<br>• Significant medical history<br>• Current medical treatments<br>• Collection of post-operative complications<br>• Collection of adverse events<br>•Patient satisfaction with Biobeat device<br>Data management<br>Patients will be identified in the study by an inclusion number.<br>Data will be entered into a secure eCRF.<br>Data collected by Biobeat patches will be transferred to a Health Data Accreditation Server (HADS).<br>Quality control:<br>The team at the Délégation à la Recherche Clinique et à l'Innovation (DRCI) at Hôpital Foch will be responsible for monitoring the study's quality control.<br>A Clinical Research Associate (CRA) from Foch Hospital, Promoter, will be in charge of monitoring the study, checking non-oppositions, quality control of collected data...

**REGULATORY**
Comité de Protection des Personnes - CPP Ile-de-France II: Favorable opinion obtained on 29/09/2020<br>CNIL: Commitment to Reference Methodology 003 (MR003) on 06/02/2017
