# Supplementary material for: Continuous remote monitoring of postoperative vital signs with the Biobeat patch: A multicenter prospective observational study
Source: PLoS One. 2026 Apr 20;21(4):e0344662. doi: 10.1371/journal.pone.0344662 (PMC13094953; doi:10.1371/journal.pone.0344662)
Supplement: Attachment — Submitted filename: 2025 - 12 - 01 - Responses to the Reviewers de PLOS.docx [file pone.0344662.s005.docx]

**Responses to the Reviewers**

We would like to thank the reviewers for their comments. Below, we address each point raised, indicating how changes have been incorporated.

In addition, we clarified the comparison of Biobeat versus routine monitoring of detection of abnormalities by including the concordance test. The tests used are explained in the statistics chapter.

**Reviewer 1**

Comment 1: Line 88: More inclusion criteria is to be provided.

Response to comment 1: We specified more precisely the inclusion criteria as follows: "Adults (≥ 18 years of age) undergoing major surgery (digestive, gynecological, orthopedic, or urological surgery with expected durations of > 2 hours and a postoperative stay of ≥ 2 nights) were eligible.”

Comment 2: Line 151: This statement should not be placed in the statistical analyses section but a subtitle under ‘sample size’. The statement could be improved. e.g. We aimed to recruit 114 participants to ensure that approximately 100 would be evaluable, accounting for an anticipated attrition rate of about 12–13%. This sample size was considered sufficient a priori for a pilot study.

Response to comment 2: We have created a specific section on the number of patients to be included intitled “Sample size”. In addition, the term “pilot study” has been removed as it creates confusion, as pointed out by Reviewer 2.

Comment 3: Line 164: The sentence ‘Paired nominal data were compared using the McNemar’s test’ requires revision. e.g. Paired nominal data were compared using McNemar’s test.

Response to comment 3: Changes have been made as suggested by the reviewer.

Comment 4: Table 1 Age, years, BMI: Indicate what the figures refer to.

Comment 5: Line 185: To use symbols to indicate number (percentage) and median [25th and 75th percentiles] {minimal and maximal values} in the table and denoted in the table footnote.

Response to comments 4 and 5: We have modified Table 1 using symbols and footnotes to summarize the main characteristics of the patients.

*: Categorial variables are presented as number (percentage)

**: Continuous variables are presented as median [25th-75th percentiles] {minimal value - maximal value}.

Comment 6: Table 2: n(%) is to be placed on top of the table after the column variable name.

Response to comment 6: This has been done as suggested.

Comment 7: Table 3, Line 246: { } is to be replaced with ( ). The statistical test is to be denoted in the table footnotes.

Response to comment 7: Table 3 has been redone in accordance with this comment and that of the Reviewer 2.

Comment 8: S4 Figure 1: The figure is inaccessible.

Response to comment 8: We apologize for the incorrect naming of the attached files (Supporting Information).

The corrected version includes the following changes:

S1 Appendix: Protocol Summary

S2 Appendix: Ethical Committee Approval

S3 Appendix: Strobe checklist

S4 Figure: Biobeat sensor and its positioning [from Paternot et al. F1000Res. 2021;10:622. doi: 10.12688/f1000research.54781.2 with permission]

Comment 9: The manuscript contains a few typographical/grammatical errors and would benefit from thorough proofreading.

Response to comment 9: The manuscript has been reviewed, and we hope that errors have been corrected.

**Reviewer 2**

General comment: The paper reports the validation of the Biobeat device in postoperative patients. The paper is overall well written. However, there are sections, mostly related with the protocol, which are not clear. For example, I was not able to understand if this is actually a prospective study, or a secondary study that came up later or in parallel. The study protocol is a clinical protocol, while this study is about the Biobeat. Please find below a list of specific comments.

to general comment: This is a prospective clinical study. We acknowledge that this was not sufficiently clear in the manuscript. The term “prospective” has been added to the title, abstract, and end of the Introduction section.

However, we believe that the chapter “Study design, ethics approval and setting” provides a sufficient description of the method, especially since we have provided a summary of the protocol (S1 Appendix) and the advice of the ethics committee (S2 Appendix).

Finally, this protocol is a clinical study, even though it concerns medical equipment, as it is in accordance with French legislation at the time of registration.

Comment 1: - Comments related to how the study protocol is presented:

--I suggest the authors to add a few details of the population within the paper and not only in the supplementary material. For example, what type of surgery was considered.

--The study has, as a primary objective, the estimate of the proportion of patients with severe hypotension, and several secondary objectives. Is this study related to only the secondary objectives? Or, the results of the study protocols are already published and this is a retrospective (and not prospective) analysis? I highly suggest authors to clarify the context.

--The sentence "We aimed to recruit patients to yield evaluable participants, which is a sufficient number a priori for a pilot study" is not scientifically supported. Why pilot study? The statistical analysis was already done. Again, are authors reusing data collected for another study? If so, the narrative must be completely changed!

-I am not clear with the protocol. It is said that the number of nights for the post-operative stay was >=2. However, the monitoring was done after ward admission for 72 hours. Was the monitoring concluded by the research assistants if the number of nights was exactly 2 (so, less than 72 h)?

Response to comment 1:

--As suggested also by Reviewer 1, we specified more precisely the inclusion criteria as follows: "Adults (≥ 18 years of age) undergoing major surgery (digestive, gynecological, orthopedic, or urological surgery with expected durations of > 2 hours and a postoperative stay of ≥ 2 nights) were eligible.”

-- We were awkward in describing the outcomes. The revised version is as follows: “The primary outcome was the proportion of patients with major hemodynamic abnormalities (MBP < 60 mmHg) using the data obtained from the Biobeat patch. The secondary outcome was the proportion of patients with vital sign abnormalities at several thresholds, either through nurse monitoring or using the study device. Other secondary outcomes concern the lack of data collection and artifact frequency (technical performance), the occurrence of postoperative complications, evaluation of ease of sensor installation, and evaluation of patients' acceptability and post-removal skin abnormality.”

-- This comment is similar to comment 2 from Reviewer 1. We created a specific section concerning the number of patients to be included in the study. The term “pilot study” has been removed as it creates confusion as pointed by the Reviewer and added the fact that the number of patients to be included was choose arbitrary: “We aimed to recruit 114 participants to ensure that approximately 100 would be evaluable, accounting for an anticipated attrition rate of about 12–13%. This number was chosen from a study published by Liem et al., who reported that postoperative hypotension (for example MBP less than 60 mmHg) occurred in 8% of the patients [17].” This was specified in the protocol (see S1 Annex) but was omitted from the text. As this was a prospective clinical study, the number of patients to be recruited was planned before the study began.

- The paragraph concerning the study duration is poorly worded. We decided on a minimum duration (see inclusion criteria) but not an upper limit of the intervention. The corrected paragraph is as follows: “Monitoring analysis began upon ward admission and was planned for at least 48 h unless terminated early due to patient withdrawal or thoracic computed tomography scan, or magnetic resonance imaging necessity. The research assistants checked for adverse events daily and removed the patch at the conclusion of the study.”

Comment 2: Please provide the sampling rate and amplitude resolution of the device.

Response to comment 2: We found no data on Biobeat’s sampling frequency or amplitude resolution. Similar wearable PPG devices sample raw signals at 50–100 Hz (based on reviews of PPG technology in wearables) to capture pulse waves, with an amplitude resolution of 12–16 bits for a sufficient dynamic range in light intensity measurements. Consequently, a scientifically rigorous and precise answer regarding the sampling and amplitude resolution specifications of the Biobeat device cannot be provided.

Comment 3: Please provide whether the experts validated the artifacts automatically detected or not. From the description "four experts reviewed the tracings to identify other artifacts", it seems they only added additional artifacts. However, from Table 2, the artifacts from clinical inspection were always lower and automatic rules. Please clarify.

Response to comment 3: The search for artifacts was conducted in two ways: by comparing values with threshold values (mathematical" evaluation) and clinical evaluation, as described in the manuscript. The "mathematical" evaluation considers each value and can isolate abnormal values (i.e., a single value between two periods where this variable is not measured), while the clinical evaluation considers all the data and can therefore detect an anomaly based on the context (evolution of the variable). This explains, at least in part, the observed results. We have changed our text to explain this point as follows: “The numerical evaluation takes each value into account and can isolate abnormal values (i.e., a single value between two periods where this variable is not measured), while the clinical evaluation considers all the data and can therefore detect an anomaly based on the context (evolution of the variable). This explains, at least in part, the results observed.”

Comment 4: In the sentence "Biobeat does not include an electrocardiogram and therefore measures PR and not HR [11]", is [11] an appropriate citation? It looks as a study in a similar domain but not necessarily supporting that you cannot estimate HR without ECG. Please, clarify.

Response to comment 4: We apologize for using reference [11], which is not appropriate for this study. We clarified this point at the end of the Biobeat presentation (Introduction section): “Biobeat does not record HR which is calculated from an electrocardiogram but PR which is derived from pulse wave signals, obtained via PPG.” No reference is needed to support this statement.

Comment 5: How the time of measurements by the nurses was recorded?

Response to comment 5: This is specified in the corrected version: “Demographic data and nurse measurements with their measurement times were extracted from hospital records.”

Comment 6: Double check the minimal resolution of the images for publishing with this journal. On my laptop, the image "S4 Fig 1 - Riviere - BioBeat device" looks rather course. Figure 3 looks course too.

Response to comment 6: The resolution of the images was checked. Figure 3 has been redrawn.

Comment 7: Is there a typo in Fig. 5? The CI for Temp is all negative but the bias is positive.

Response to comment 7: We apologize for this typographical error. Ci for Temp is [-0.4; 2.4]. Figure 5 has been modified accordingly in the revised manuscript.

Comment 8: I am not understanding the values in curly brackets in Table 3. What is "Number of observations are reported as {}"? Observation=patients?

Comment 9: Table 3 states reporting the number of patients. However, the percentages are not summing up to 1. I think authors are reporting values across measurements and not patients.

Responses to comments 8 and 9:

Table 3 shows the number of patients and their percentage relative to the number of patients for whom the data were available. The sum is not 100% because it is a "Russian doll" presentation, which is a nested structure. Furthermore, the same patient may be counted in several categories; a patient may have been hypothermic and febrile during the monitoring period, for example.

Table 3 has been modified significantly:

- We have simplified Table 3 to improve clarity. We removed the data concerning the percentage change in blood pressure relative to the value measured before anesthesia, since these data did not add much value.

- We have introduced agreement between by Biobeat and/or by nurses’ measurements of hemodynamic, respiratory, and temperature abnormalities for each threshold.

This leads to two statistical analyses as explained in the statistical chapter: **“**For binary paired data, agreement between nurse and Biobeat classifications was evaluated using Cohen’s kappa coefficient, which quantifies agreement beyond chance but is sensitive to the distribution of positive and negative categories. To assess whether discordant pairs favored one method (whether nurse or patch produces significantly more positive, or negative results than the other), McNemar’s test with continuity correction was applied. When the total number of discordant pairs was <10, the exact McNemar test was used instead.”

This leads to results resulting from these tests: “Agreement between nurse and Biobeat detection of these abnormalities was generally low, except for MBP < 60 mmHg, where Cohen’s kappa suggested fair agreement (0.30 [-0.04;0.64]). Severe hypotension (MBP <60 mmHg, the primary objective) was not more frequently detected with Biobeat than with nurse surveillance (*P* = 0.07, McNemar test) but this was the case when considering the threshold of 65 mmHg (*P* = 0.01). Biobeat also showed higher rates of discovery for PR ≥ 100 beats/min (*P* < 0.001), RR > 20 breaths/min (*P* < 0.001), SpO2 up to > 90% (*P* < 0.001), and temperature <36,8° or >38 °C (*P* < 0.001).”

Comment 10: What are the "1.1% serious issues" in sensor installation?

Response to comment 10: We modified the paragraph: “Sensor installation was straightforward in 76 cases. Minor issues occurred in 10 patients, and moderate issues occurred in three patients. The sensor was not recognized by the platform in one case which prevented its configuration in the PACU; the configuration was subsequently completed when the patient was in the surgery department.”

Comment 11: What data support "the sensor outperformed conventional monitoring in detecting vital sign abnormalities" in this study?

Response to comment 11: This is supporting by data presented in Table 3 and analysis using McNemar test: Biobeat identifies more patients with postoperative hypotension (<65 mmHg), tachycardia (≥100 beat/min), polypnea (>20 breaths/min), oxygen desaturation (SpO2 < 90%) and temperature (<36,8°ot >38°) than nurse. We have modified the first paragraph of the Discussion section as follows: “This study of the Biobeat wearable sensor in postoperative patients, conducted without human intervention, produced mixed results: significant data loss, few artifacts, and varied agreement with nurse measurements across parameters. However, the sensor allows for more frequent detection of several vital sign abnormalities than conventional nursing monitoring.”

We also emphasized that the population studied was "not very serious" in the chapter. “Ability to detect vital sign abnormalities relative to standard care” as follows: **“**This was not the case in our study, as shown by the low number of patients, or even their absence, when considering the occurrence of very severe hypotension (MAP < 60 mmHg), bradycardia (PR ≤ 40 beats/min), bradypnea (RR < 8 breaths/min), or hyperthermia (temperature > 39°C).”

Comment 12: - I am not sure what authors mean with "real-world" study. The clinical protocol refers to a clinical study (which is of course real). In most studies involving wearables, "real-world" means "into the wild" ie outside the clinical environment, where there is little to no control of what the person is doing during monitoring. Please, clarify.

Response to comment 12: This term was indeed used inappropriately. We would like to emphasize that the patients were hospitalized in a conventional unit with standard nursing supervision. Wearing the Biobeat patch did not alter their care in any way; the only specific element was that a research assistant checked for adverse events daily. We preferred to remove the term “real-world” in the corrected version, as it was indeed a source of confusion and did not provide any relevant information to the reader. Instead, we introduced the term:” study conducted under usual clinical conditions” in the abstract, the first paragraph of the Discussion and in the first paragraph do the

Strengths and limitations section and finally in the Conclusion.

Comment 13: I think authors should give some additional details to better understand "The simplest hypothesis is that patients move beyond the router’s 10-meter range.". For example: Does the patch have storing capabilities? Does the patch contain a log of connections/disconnections?

Response to comment 13: The Biobeat patch has an internal memory for temporary data storage when the device is offline or disconnected from the app and the cloud. Once reconnected, the stored data were uploaded to the Biobeat cloud platform. Since we used automated data transfer to a national storage site rather than to the Biobeat storage site to meet data protection requirements, this may explain part of the data loss.

We do not have a log of connections/disconnections, as we did not anticipate these issues.

Interestingly, Helmer et al. recently reported that Biobeat wrist-worn devices yielded valid data only for 61.5% of the monitored time (median: 57.6%; range: 20.1-78.3%) although each patient room was equipped with a dedicated router, and additional routers were strategically placed in the corridors to ensure optimal signal coverage across the ward. To explain these results, the authors cited battery limitations, incorrect positioning of the wrist-worn monitors, and the device’s internal algorithm rejecting values of poor signal quality. They consider other reasons, such as failures of data transmission, processing, or storage errors, to be unlikely. Furthermore, valid data available for SpO2 were obtained in only 45.1% of recordings.

We have completely rewritten the paragraph on data loss: “A key finding was the substantial loss of data, encompassing both the complete absence of all variables and partial gaps in specific variables. This issue has been well documented. Hara et al. highlighted similar challenges in wireless monitoring [27], while Merschel et al., using a PPG-equipped smartwatch (Cardio Watch 287; Corsano Health BV), reported analyzable data ranging from 34% to 100%, which decreased during activity [28]. Using Biobeat chest patches, Belliveau et al. used Bluetooth and patients’ phones and noted that all participants experienced at least one 2-hour data gap [13]. Helmer et al. used Biobeat wrist-worn devices and equipment of patient room and corridors with dedicated routers and reported valid data for 61.5% of the monitored time (range: 20.1-78.3%) [29]. In the absence of data transfer monitoring, we can only speculate on the reasons for these data losses throughout the transmission chain: sensor detachment (possibly from sweating), device’s internal algorithm rejecting values of poor signal quality, router failure or battery limitation, disrupted cloud transfer, patient displacement beyond the area covered by the router.”

Comment 14: - What is LoA at line 307?

Response to comment 14: LoA is “Limits of Agreement”. The term has been modified in the revised version. The 95% CI of the agreement limits is presented in Figure 5 for each variable.

Comment 15: What was the percentage of PR measurements which were within +/- 10 bpm? Figure 5 reports the limits where approximately 20 bpm. So, is it the Biobeat similar to Empatica E4 and other devices if the same threshold is considered?

Response to comment 15: There are many methods to compare the measurements obtained by the two devices. We chose the Bland–Altman analysis and Clark error grid representation. As presented in Figure 5, the limits of agreement for PR were -17.6 and 16.5 bpm. Xu et al. reported that 74.5% of their HR measurements from the Empatica E4 wrist sensor were within ±10 bpm of the reference standards. We found a similar result (73.7%). The revised version is as follows: “A quite similar value, 73.7%, was found in our study.“

Comment 16: The sentence "Finally, the Biobeat® performance has likely improved since the period of this study" should be removed. It does not add anything to the study and it is not supported either by additional references.

Response to comment 16: We have deleted this sentence, as suggested.

Comment 17: -Typos:

--Do not reinitialize acronyms (for example, PACU): Done.

--Artifacts or Artifacts Please uniform the use: We keep the word “artifact”.

-->110 { 90}: Table 3 has been modified.

--hadn’t -> had not
