# Supplementary material for: Continuous remote monitoring of postoperative vital signs with the Biobeat patch: A multicenter prospective observational study
Source: PLoS One. 2026 Apr 20;21(4):e0344662. doi: 10.1371/journal.pone.0344662 (PMC13094953; doi:10.1371/journal.pone.0344662)
Supplement: Attachment — Submitted filename: renamed_7d39b.docx [file pone.0344662.s006.docx]

**Response to the Reviewer 3**

General comment

The current manuscript reports the performance of a remote monitoring device. The reviewer was not part of the previous round(s) of reviews. The writing is generally good. The reviewer has the following comments.

Response to the general comment:

We thank the reviewer for their comments and have amended the manuscript accordingly. However, some comments relate to formatting and these sometimes conflict with the journal's recommendations. This is noted below.

Some phrasing has been revised.

Deleted elements in the modified text are in red and strikethrough; added/replacement text is highlighted in blue.

Comment 1) Line 63: Equipment can be cited in-text with the company name and location without its website.

Response to Comment 1): Modification has been made as requested: “The Biobeat system (Biobeat Technologies Ltd., Petah Tikva, Israel) …”

Comment 2) Introduction: Before introducing the Biobeat sensor, please expand the review of existing products with similar functionalities. Currently, only lines 59-62 satisfy this requirement.

Response to Comment 2): Introduction: We have modified the introduction to address your comment: “Since the early 2000s, the rise of broadband Internet, Wi‑Fi, and Bluetooth has transformed remote patient monitoring from occasional telemedicine experiments into structured systems capable of continuously measuring and transmitting patients’ clinical parameters such as blood pressure (BP), heart rate (HR), pulse rate (PR), respiratory rate (RR), peripheral oxygen saturation (SpO2), and temperature. Numerous wearable devices now enable the simultaneous capture of multiple vital signs and transmit them in real time to monitoring platforms or electronic medical records. A recent narrative review by Bignami et al. described nine clinically validated multiparametric monitoring devices [5]. They show that, although the dividing line remains debatable, some are primarily designed for in‑hospital use (CheckPoint Cardio CPC12S [6], Lifetouch [7], Portrait Mobile [8], Radius VSM [9], SensiumVitals [9], VitalPatch [10]) while others are aimed more at out‑of‑hospital settings, particularly prehospital environments and the home (BioButton [11], CardioWatch 287-2 [12], C-Med Alpha [13].”

Comment 3) Lines 81-83: Published guidelines should be cited as formal references.

Response to Comment 3): We believe that you are referring to the protocol and to the ethics committee agreement, which are on lines 81-83 of the submitted PDF. However, if these items are cited as formal references, the reader will no longer have access to them. That is why we prefer to keep the current format, but perhaps we have misunderstood your comment.

Comment 4) The study design should describe the basic settings in the main text. You cannot put everything in the appendices.

Response to Comment 4): In response to this comment, we have modified the text:

- we specified more precisely the inclusion criteria: « Adults (≥ 18 years of age), classified American Society of Anesthesiologists physical status 1 to 3, undergoing major surgery (digestive, gynecological, orthopedic, or urological surgery with expected durations of > 2 hours and a postoperative stay of ≥ 2 nights) were eligible regardless of their comorbidity.”

- we have retained a chapter entitled "Study procedure" which covers both monitored surveillance and nursing surveillance.

This approach provides readers with access to essential protocol elements without requiring consultation of the Appendix.

Comment 5) More basic information should be provided for patients.

Response to Comment 5): We have clarified this in the corrected version: “Written informed consent was obtained from all participants prior to enrollment, after the study protocol (including duration, methods, and outcome assessment) had been explained and the device presented.”

Comment 6) Please provide all figures using commonly used formats, such as PDF. Figure S4 is not readable. Why is it not part of the normal figures in text? Figures 1-5 can be read correctly.

Response to Comment 6): According to PLOS One formatting requirements, figures must be submitted in TIFF or EPS format only ([**https://journals.plos.org/plosone/s/figures#loc-file-format**](https://journals.plos.org/plosone/s/figures#loc-file-format)).

Regarding Figure S4: This figure is present in the list of submitted files and in the complete PDF (page 56/96) that was returned to us for confirmation of article submission acceptance. Figure S4, which illustrates the Biobeat patch, was originally Figure 1 in the initial submission but was moved to supplementary materials following a directive from the journal editorial board received on August 5, 2025: "Please note that you must upload a completed CONSORT flowchart as Figure 1 of your manuscript." We were therefore obliged to follow this requirement. Since this figure logically belonged in the device description section, the supplementary materials format was the only appropriate placement.

Comment 7) Line 102: All abbreviations should be spelled out when being used for the first time in the text (there are other similar incidents in the manuscript). Citations are required for PPG.

Response to Comment 7): We have verified that each abbreviation is spelled out in full when first mentioned.

The manuscript already included a reference to the use of PPG for cardiovascular assessment (lines 62-63) however, we have added a reference for general PPG applications. We have modified the text as follows: “Devices such as Biobeat, CardioWatch 287-2 [14] and Checkpoint Cardio’s CPC12S [6] also approximate ~~blood pressure (~~BP~~)~~ using photoplethysmography (PPG) [15], which is a widely adopted method for portable cardiovascular assessment [16].”

Comment 8) Line 124: Web sources need to be cited as a formal reference, not just an in-text link.

Response to Comment 8): Modified as requested. The platform name is now presented in the text, and the website is cited as a formal reference.

Comment 9) Spell out “hours”

Response to Comment 9): Modified as requested. We have applied this change consistently throughout the entire manuscript.

Comment 10) The subsection of “outcomes” is not necessary and can be incorporated into the results section. The subsection of “sample size” is not necessary either and can be incorporated into “patient population”.

Response to Comment 10): The STROBE Statement explicitly requires "Clear definition of all outcomes" and "Study size: Explanation of how the study size was determined." This is why our manuscript retains these subsections in accordance with reporting guidelines.

Comment 11) “Statistical Analysis”: All named methods mentioned in this section should be briefly explained and cited.

Response to Comment 11): This paragraph has been revised as requested: “For continuous paired measurements, nurse-recorded values and the closest corresponding Biobeat values were compared using the Bland-Altman method for repeated measures, which assesses agreement between two quantitative methods by plotting differences against means and estimating within- and between-subject variance–adjusted limits of agreement [32]. Comparisons were not performed if the interval between these measurements was greater than 5 minutes. These paired measurements were also evaluated for clinical accuracy using Clarke Error Grid analysis, originally developed for self-monitoring of blood glucose [33]. A standard scatter plot of the sensor and nurse recordings was generated and divided into five zones (A–E). Zones A and B indicate acceptable agreement, and zones C–E indicate increased discrepancies. Clarke Error Grid zones were defined according to the authors’ clinical judgment and criteria from prior validation studies [9,34–36]. For binary paired data, agreement between nurse and Biobeat classifications was evaluated using Cohen’s kappa coefficient, which quantifies agreement beyond chance but is sensitive to the distribution of positive and negative categories [37]. To determine whether discordant pairs favored one method (whether nurse or patch produces significantly more positive or negative results than the other), McNemar’s test with continuity correction was applied [38]. When the total number of discordant pairs was <10, the exact McNemar test was used instead.”

Comment 12) Table 1: This is not part of the results. This should be part of the “patient population” information. See 5) above.

Response to Comment 12): We have incorporated key demographic and clinical information into the population description: “Adults (≥ 18 years of age), classified American Society of Anesthesiologists physical status 1 to 3, undergoing major surgery (digestive, gynecological, orthopedic, or urological surgery with expected durations of > 2 hours and a postoperative stay of ≥ 2 nights) were eligible regardless of their comorbidity.” However, we have retained a summary table of principal patient characteristics, as is standard practice in clinical research reporting.

Comment 13) Figure 2: This should be part of “data collection”.

Response to Comment 13): Please see our response to your comment 6.

We were required to follow the directive received from PLOS on August 5, 2025: "Please note that you must upload a completed CONSORT flowchart as Figure 1 of your manuscript."

This requirement necessitated the repositioning of all subsequent figures after Figure 1.

Comment 14) Figure 3 is missing?

Response to Comment 14): Figure 3 was present in the complete PDF that was returned to us for confirmation of article submission acceptance (page 50/96). We apologize for any confusion caused by the apparent reversal between Figures 3 and 4 (in the PDF).

Figure 3 is page 48 of the present version.

Comment 15) “Biobeat Sensor Artifacts”: How did you define an artifact? There must be an objective definition. This should be defined in the Method section first.

Response to Comment 15): We provided objective definitions in the first sentence of the Statistical analysis subsection. Recognizing this was suboptimal placement, we have relocated this text to the end of the "Data Collection" section for clarity.

Comment 16) “Biobeat Sensor Artifacts”: If the percentage is too small, increase the effective digits. It does not make sense to have a number, but the percentage is zero.

Response to Comment 16): We have revised the percentages to be expressed per 1,000. Thank you for pointing out this reporting issue.

Comment 17) Figure 5 is missing?

Response to Comment 17): Figure 5 was present in the complete PDF that was returned to us for confirmation of article submission acceptance (page 50/96). We again apologize for any confusion caused by the reversal between Figures 3 and 4.

Figure 5 is page 50 of the present version.

Comment 18) The font of section headings is a little confusing. Please add section numbers (1.1, 1.2, etc.)

Response to Comment 18): We have followed the formatting recommendations specified by PLOS One in their official guidelines (PLOSOne_formatting_sample_main_body.pdf).

Comment 19) Some of the “discussion” should be moved to results, as the discussion section is further explanation/analysis of results. No new data should appear in the discussion.

Response to Comment 19): While we respectfully acknowledge your concern, we note that our discussion section includes recalled results primarily to compare them with findings in the literature, which is standard practice. However, we have removed the sentence "~~A similar value (73.7%) was found in our study~~" because this metric was not analyzed using the same comparative methodology for other variables.
