# Supplementary material for: Continuous remote monitoring of postoperative vital signs with the Biobeat patch: A multicenter prospective observational study
Source: PLoS One. 2026 Apr 20;21(4):e0344662. doi: 10.1371/journal.pone.0344662 (PMC13094953; doi:10.1371/journal.pone.0344662)
Supplement: Attachment — Submitted filename: renamed_f7b58.docx [file pone.0344662.s007.docx]

We sincerely thank the Editorial team at PLOS ONE and the reviewers for their thoughtful evaluation of our manuscript. This response details our point-by-point replies to the reviewer’s comments and summarizes the modifications made to the manuscript. Deleted sentences are in red and crossed out in the marked text, while added sentences are in blue.

In addition, some words and sentences have been modified to make the text flow better; they are reported in blue in the marked file.

# **Reviewer #4**

Comment 1: 16% of the SpO₂ data were missing, which limits accurate evaluation of oxygen saturation trends. This gap may underestimate the device’s performance in detecting hypoxemia.

Response to Comment 1: We appreciate this important observation and recognize that data completeness is a key metric of technical performance. We have substantially revised the manuscript to clarify the nature, quantification, and explanation of SpO2 data loss.

First, we modified the chapter “Biobeat Monitoring System Data collection” as follows: "Of 64,088 expected simultaneous multi‑parameter physiological measurements at 5‑minute intervals, 28,090 (43.8%) were missing, while 29,514 contained complete data for all variables. Among the 6,484 incomplete serial measurements, 89.6% lacked SpO2, 11.1% lacked MBP, and 8.9% lacked RR. PR and temperature were missing in only 7 and 3 instances, respectively. Overall, SpO2 was the most frequently absent variable, occurring in 16.1% of all obtained serial measurements, either in isolation or alongside other missing parameters. Fig 3 illustrates an example of complete monitoring and incomplete data with periodic gaps."

Second, we have completed the chapter on "Data loss" in the Discussion section as follows: “Finally, one particular point concerns SpO2, which emerged as the parameter most frequently missing when data transmission has been satisfactory. SpO2 is intrinsically difficult to measure accurately because the arterial pulsatile component of PPG represents only a small fraction of the overall optical signal and is easily contaminated by noise. Motion artifacts, low peripheral perfusion, vasoconstriction, ambient light, and sensor misplacement can all distort the PPG waveform and lead to erroneous SpO₂ estimates which are rejected by the device [41]”.

Comment 2: The median recording period of about 64 hours limits insights into long-term performance, connectivity stability, and patient adherence.

Response to Comment 2: We agree that the 64-hour median monitoring period represents a limitation and appreciate the reviewer's point about long-term performance. However, we believe this duration is appropriate for the study's objectives and is comparable to the existing literature. We have substantially revised the manuscript to contextualize this timing. This is discussed in the chapter Strengths and limitations as follows in the revised manuscript: “Third, we included patients with an expected postoperative hospital stay of ≥ 2 nights. This criterion was based on evidence that the majority of postoperative complications (approximately 60%) occur within 1–3 days after surgery, as demonstrated in a cohort study of patients undergoing intra-abdominal operations [55]. However, future prospective studies should evaluate extended monitoring in patients requiring prolonged postoperative supervision (e.g., those undergoing complex surgeries or with severe comorbidities), with monitoring extending at least through the first week post-discharge.”

# **Reviewer #5**

Comment 1: First‑mention definitions of all abbreviations and clearer referencing of PPG methodology.

Response to Comment 1:

We have verified that all abbreviations are defined when they first appear and we gave a brief overview of PPG technology: “Blood pressure can be measured using photoplethysmography (PPG) which is a non-invasive optical technique that detects changes in microvascular blood volume through analysis of light absorption variation. The direct current (DC) component represents baseline tissue absorbance, whereas the alternating current (AC) component reflects cardiac-synchronous arterial blood volume changes. This enables reliable extraction of SpO2, PR, and RR through validated algorithms, with the perfusion index (AC/DC ratio) serving as a clinical indicator of peripheral perfusion status. Waveform morphology analysis permits non-invasive assessment of arterial stiffness through pulse wave features [13]. Wearable devices such as Biobeat [14] and CardioWatch 287-2 [15] have demonstrated blood pressure estimation capabilities through PPG analysis in controlled, calibrated settings, though pulse transit time-based BP estimation encounters substantial technical limitations, including pre-ejection period contamination and insufficient signal information content, with long-term ambulatory accuracy remaining inadequately characterized. The Checkpoint Cardio CPC12S attempted blood pressure measurement through PPG but demonstrated unacceptably low accuracy in validation testing, exemplifying the current limitations of cuffless BP estimation from optical signals [5].”

Comment 2: Enhanced introduction summarising comparable wearable systems.

Response to Comment 2:

We have modified this paragraph: “Numerous wearable devices can capture multiple vital signs simultaneously and transmit data in real time to monitoring platforms or electronic medical records. In a recent narrative review, Bignami and colleagues identified nine clinically validated multiparametric monitoring systems. Although distinctions are not absolute, several devices are designed primarily for in‑hospital use (CheckPoint Cardio’s CPC12S, Lifetouch, Portrait Mobile, Radius VSM, SensiumVitals, and VitalPatch [5-9]), whereas others are oriented toward out‑of‑hospital applications, particularly prehospital and home monitoring (BioButton, CardioWatch 287‑2, and C‑Med Alpha [10-12]).”

Comment 3: Improved description of study procedures and inclusion criteria, now appropriately integrated into the main text. Better clarity regarding artefact definitions and their placement within the Data Collection section.

Response:

We have modified the Data Collection section and the Outcome section to respond to this comment.

- Revised Data Collection section: “Each patient was assigned a unique identifier at sensor installation. Demographic data and nurse measurements with corresponding timestamps were extracted from hospital medical records. Although Biobeat real-time data display was available, it was not utilized during this study. Biobeat data were retrieved post-study from the Instamed company [24]. We aimed to obtain synchronous measurements of vital signs (PR, MBP, RR, SpO2, and temperature) at 5‑minute intervals. Artifact frequency (secondary outcome indicating technical performance) was particularly examined, with objective definitions based on previously published criteria. Artifacts were defined as values exceeding 50% deviation from the prior reading (unless followed by a ±25% recovery value) or falling outside physiological ranges (e.g., PR <5 or >250 bpm; systolic BP <20 or >300 mmHg; SpO2 change ≥8% between consecutive readings) [25-28]. After numerical analysis, four independent experts reviewed the tracings to identify additional artifacts not captured by the automated criteria.”

- Revised Outcome section: “The primary outcome was the proportion of patients with major hemodynamic abnormalities defined as mean blood pressure (MBP) <60 mmHg, detected by the Biobeat patch. The secondary outcome was the proportion of patients with vital sign abnormalities at various predefined thresholds as detected by either nurse monitoring or the Biobeat device. Other secondary outcomes include data loss and artifact frequency (technical performance), incidence of postoperative complications, assessment of sensor installation ease, and evaluation of patient acceptability and post-removal skin reactions.”

Minor Suggestions: A brief tightening of certain sentences in the Discussion could further enhance clarity, although the current version is acceptable.

Consider removing any residual repetition regarding consent and data transmission procedures.
Response to minor suggestions:

We reviewed the Discussion and tried to make the text clearer and removed repetition.
